# CuAS: a database of annotated transcripts generated by alternative splicing in cucumbers

**DOI:** 10.1186/s12870-020-2312-y

**Published:** 2020-03-18

**Authors:** Ying Sun, Quanbao Zhang, Bing Liu, Kui Lin, Zhonghua Zhang, Erli Pang

**Affiliations:** 1 MOE Key Laboratory for Biodiversity Science and Ecological Engineering and Beijing Key Laboratory of Gene Resource and Molecular Development, College of Life Sciences, Beijing Normal University, No 19 Xinjiekouwai Street, Beijing, 100875 China; 2 Key Laboratory of Biology and Genetic Improvement of Horticultural Crops, Ministry of Agriculture, Institute of Vegetables and Flowers, Chinese Academy of Agricultural Sciences, Beijing, 100081 China

**Keywords:** Cucumber, Alternative splicing, Isoform-level function, Isoform-level features, Tissue-specific alternative splicing events

## Abstract

**Background:**

Alternative splicing (AS) plays a critical regulatory role in modulating transcriptome and proteome diversity. In particular, it increases the functional diversity of proteins. Recent genome-wide analysis of AS using RNA-Seq has revealed that AS is highly pervasive in plants. Furthermore, it has been suggested that most AS events are subject to tissue-specific regulation.

**Description:**

To reveal the functional characteristics induced by AS and tissue-specific splicing events, a database for exploring these characteristics is needed, especially in plants. To address these goals, we constructed a database of annotated transcripts generated by alternative splicing in cucumbers (CuAS: http://cmb.bnu.edu.cn/alt_iso/index.php) that integrates genomic annotations, isoform-level functions, isoform-level features, and tissue-specific AS events among multiple tissues. CuAS supports a retrieval system that identifies unique IDs (gene ID, isoform ID, UniProt ID, and gene name), chromosomal positions, and gene families, and a browser for visualization of each gene.

**Conclusion:**

We believe that CuAS could be helpful for revealing the novel functional characteristics induced by AS and tissue-specific AS events in cucumbers. CuAS is freely available at http://cmb.bnu.edu.cn/alt_iso/index.php.

## Background

Alternative splicing (AS) is an important post-transcriptional process by which multiple transcripts are generated from a single gene. It plays critical roles in adaption to the environment, development, and tissue specificity [1–4]. Additionally, it increases the functional diversity of proteins [2].

Since the first discovery of AS 40 years ago [5], an increasing number of alternatively spliced genes have been reported. With the development of sequencing technology, it has been found that AS is apparently highly pervasive in eukaryotes. Recently, based on RNA-Seq data, 95% of human genes [6] and 61% of *Arabidopsis* genes [7] were reported to undergo AS. In addition, the functions of AS have been investigated. Emerging experimental evidence indicates that AS can regulate the following properties of proteins: 1) binding to other proteins and nucleic acids [8], 2) the localization of proteins according to localization signals [9], 3) enzymatic properties [10], and 4) interactions with ligands [11]. Overall, AS can influence almost every aspect of protein functions [2].

Several AS databases such as ASpedia [12], VastDB [13], and DBATE [14] have been established, but these databases are for vertebrates, especially humans, and few of them address AS in plants. In plants exposed to environmental stress, many biological processes are regulated by alternative splicing [3]. With the development of sequencing technologies, the detection of AS in plants is coming of age [15]. Therefore, a database for the annotation of AS events and a retrieval system to query AS and explore the functions of alternatively spliced transcripts in plants is needed.

Here, we introduce a database of annotated transcripts generated by AS in cucumbers (CuAS) (*Cucumis sativus* L. var. *sativus* cv. 9930 and *Cucumis sativus* var. *hardwickii* PI 183967). The database provides five types of data: (1) genomic annotation, (2) AS events analysed from multiple tissues, (3) isoform features, (4) isoform functions, (5) and splicing events among tissues. The web application includes four components: an annotation database, a retrieval system, a browser, and tools. This user-friendly database will serve as a hub for revealing the functional characteristics induced by AS and tissue-specific AS events in cucumbers.

## Construction and content

**Fig. 1:**
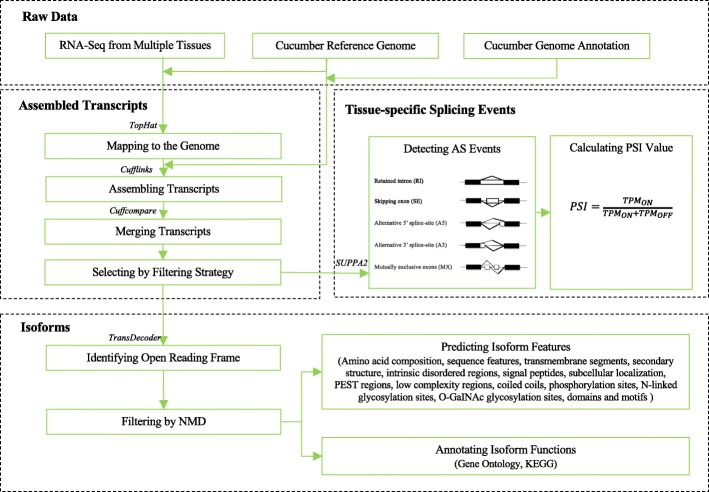
Overview of the construction of the CuAS database

The CuAS database integrates genomic annotation, AS events from multiple tissues, isoform functions, isoform features, and tissue-specific splicing events. The integration steps are shown in Fig. 1.

### Data sources

CuAS includes data from two varieties of cucumber: *Cucumis sativus* L. var. *sativus* cv. 9930 and *Cucumis sativus* var. *hardwickii* PI 183967. The genome sequences and genome annotations were collected from http://cmb.bnu.edu.cn/Cucumis_sativus_v20/. The RNA-Seq data of ten tissues from *Cucumis sativus* L. var. *sativus* cv. 9930 were downloaded from the SRA database (https://www.ncbi.nlm.nih.gov/sra/) (SRA: SRA046916), and the RNA-Seq data of seven tissues from *Cucumis sativus* var. *hardwickii* PI 183967 were obtained from the website http://cmb.bnu.edu.cn/Cucumis_sativus_v20/. The seven tissues included the roots, stems, leaves, male flowers, female flowers, fruit, and tendrils.

### Identification of alternative splicing events and isoforms

In previous research based on RNA-Seq of ten tissues from *Cucumis sativus* L. var. *sativus* cv. 9930, we assembled transcripts by using TopHat and Cufflinks [16], respectively. These sets of transcripts were then compared with the reference genome annotation file using Cuffcompare. The transcripts were divided into 12 categories according to the output of Cuffcompare. Then, the following strategies were applied to obtain high-quality transcripts [17, 18]. First, all of the transcripts with three class codes (=, j, o) (http://cole-trapnell-lab.github.io/cufflinks/cuffcompare/) were extracted from the output generated by Cuffcompare. The transcripts in the “j” and “o” classes were considered novel transcripts. Next, the novel transcripts with a single exon were removed, and we obtained an assembled cucumber transcriptome. To reduce potentially misassembled transcripts, each novel splice junction was required to be supported by at least ten reads, and each known splice junction was required to be supported by at least one read. According to these criteria, transcripts supported by certain splice junction reads were obtained. Finally, transcripts per million reads (TPM) values were calculated by using Salmon (version 0.13.0) [19], and the transcripts with TPM values of greater than or equal to one in at least one sample were used for the analysis [20]. With the implementation of a series of filters, a high-quality putative transcriptome was obtained. Based on the obtained transcripts, AS events were identified by using SUPPA2 (version 2.3) [21]. The AS events were classified into five types: retained intron (RI), skipped exon (SE), alternative 3′ splice-sites (A3), alternative 5′ splice-sites (A5), and mutually exclusive exons (MX).

To better understand the impact of differentially spliced isoforms encoded by a single gene, we used TransDecoder (https://github.com/TransDecoder/TransDecoder, version 3.0.1) to identify the candidate coding regions in the assembled transcripts. TransDecoder performs homology searches against Pfam 30.0 [22] and the UniProt database (version 2016_11) [23] to obtain supporting evidence for the open reading frames (ORFs). We selected the single best ORF for each transcript using the parameter “-single_best_orf”. If a premature termination codon was located more than 55 nucleotides from the last splice junction, the transcript was considered to be a result of nonsense-mediated mRNA decay (NMD) [24–26]. Any transcript with an ORF that is greater than or equal to 300 bp in length that did not show NMD was retained for further analysis. The same software and parameters were used for *Cucumis sativus* var. *hardwickii* PI 183967.

### Functional annotation at the isoform level

First, we performed a Blast2GO [27] analysis that assigned gene ontology terms to each isoform. Blast2GO performed a BLASTP search (E-value 1e-05) against the UniProt (release 2017_06) database. Then, the identified isoforms were mapped to reference canonical pathways in the Kyoto Encyclopedia of Genes and Genomes (KEGG) (https://www.genome.jp/kegg/, version 90.1) [28]. KAAS (KEGG Automatic Annotation Server, https://www.genome.jp/tools/kaas/) was used to assign KEGG pathways.

### Prediction of features at the isoform level

The software used for the prediction of isoform features is listed in Table 1. In total, 15 types of features were predicted, including the amino acid composition, sequence features, transmembrane segments, secondary structure, regions of intrinsic disorder, signal peptides, subcellular localization, PEST regions, low-complexity regions, coiled coils, phosphorylation sites, N-linked glycosylation sites, O-GaINAc glycosylation sites, domains, and motifs.

**Table 1:** Software used for isoform feature prediction

Feature Group	Software	Reference
Amino acid composition	EMBOSS-6.6.0	[39]
Sequence features	EMBOSS-6.6.0	[39]
Gravy	GRAVY calculator	(no warranty)
Transmembrane segments	MEMSAT 3.0	[40]
Secondary structure	PSIPRED 4.0	[41]
Intrinsically disordered regions	DISOPRED 3.16	[42]
Signal peptides	SinglP 4.0	[43]
Subcellular localization	YLoc	[44]
PEST regions	EMBOSS-6.6.0	[39]
Low complexity regions	EMBOSS-6.6.0	[39]
Coiled coils	EMBOSS-6.6.0	[39]
Phosphorylation sites	NetPhos-3.1	[45]
N-linked glycosylation sites	NetNGlyc-1.0c	[45]
O-GalNAc-glycosylation sites	NetOglyc-3.1d	[45]
Domains (Pfam)	InterProScan 5.24	[29]
Motifs (Prosite)	InterProScan 5.24	[29]

The transmembrane segments, secondary structure, and regions of intrinsic disorder were searched against the UniRef90 dataset (release 2016_01). Domains and motifs were assigned using InterProScan 5.24 [29].

### Tissue-specific splicing events

To investigate tissue-specific splicing events, the percent spliced-in index (PSI), which is a representative AS event measurement, was quantified for all AS events. The PSI measures the fraction of the mRNAs expressed from a gene that contains a specific form resulting from an AS event [30]. The reads were used to quantify transcript abundances with Salmon [19], and the PSI values [31] among tissues were calculated by SUPPA2 for all AS events.

### Prediction of gene descriptions and gene families

The functional description of the genes was provided by the AHRD tool (https://github.com/groupschoof/AHRD) based on the results of BLASTP searches against UniProt and TAIR. In regard to gene families, transcription factors (TFs), transcriptional regulators (TRs), and protein kinases (PKs) were identified by iTAK (version 1.7) [32]. While splicing-related genes were identified by OrthoFinder (version: 2.3.1) [33] against the sequences of *Arabidopsis* [34], including small nuclear ribonucleoproteins, splicing factors, splicing regulation-related proteins, novel spliceosome proteins, and possible splicing-related proteins.

### Web implementation

The web interface is implemented with PHP programming, HTML, and JavaScript. All the graphs are generated through the plug-in ECharts [35]. All the tables are in the style of Layui (https://www.layui.com/). Poshy Tip (https://github.com/vadikom/poshytip) is applied to show the position of amino acids.

## Utility and discussion

The CuAS system contains four components: an annotation database, a retrieval system, a browser, and tools (BLAST and JBrowse).

### Database overview

In total, a set of 60,643 transcripts (36,274 from *Cucumis sativus* L. var. *sativus* cv. 9930 and 24,369 from *Cucumis sativus* var. *hardwickii* PI 183967) was obtained. Based on these transcripts, 10,748 AS events (6673 from *Cucumis sativus* L. var. *sativus* cv. 9930 and 4075 from *Cucumis sativus* var. *hardwickii* PI 183967) were predicted, and 49,018 isoforms (28,588 from *Cucumis sativus* L. var. *sativus* cv. 9930 and 20,430 from *Cucumis sativus* var. *hardwickii* PI 183967) were retained for the analysis of features and functions. Isoform functions were annotated with Gene Ontology [36] and KEGG [28] terms. Regarding isoform features, 15 types of features were predicted. In addition, the PSI values were quantified for all AS events (see Construction and Content).

### Web interface

**Fig. 2:**
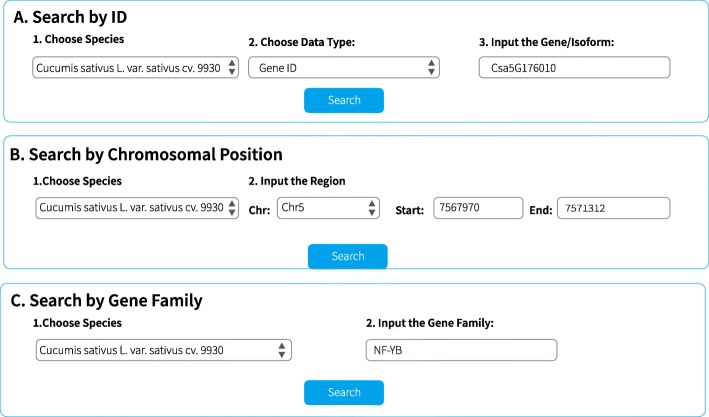
Search tools of CuAS

The CuAS web-interface provides access to genomic annotation, functional annotation at the isoform level, features at the isoform level, and tissue-specific AS events. The data can be queried using three input formats: ID (gene ID/isoform ID/UniProt ID/gene name), chromosomal position, and gene family (Fig. 2, e.g., *Csa5G176010*). These input data can be used to search AS events among tissues and their relevant annotations.

**Fig. 3:**
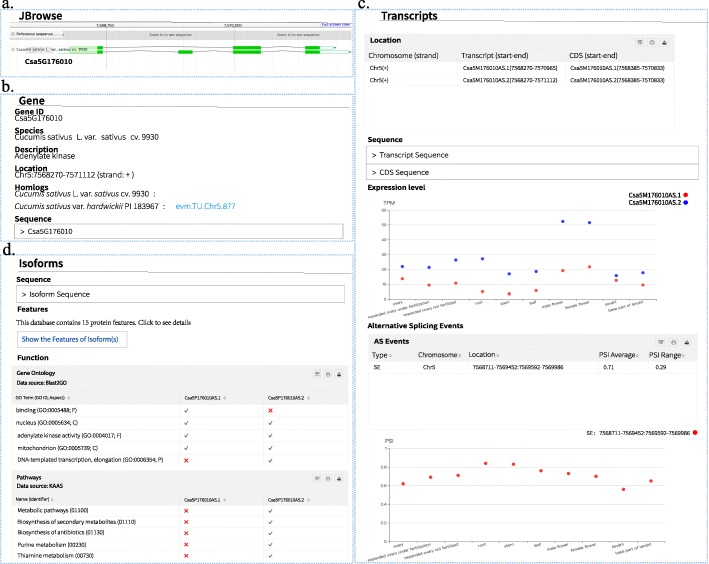
Web interface of CuAS. (**a**) JBrowse. (**b**) At the gene level. (**c**) At the transcript level. (**d**) At the isoform level

Search results are categorized and visualized on the results page, as illustrated in Fig. 3 by using the example of *Csa5G176010*. The structures of the two transcripts encoded by *Csa5G176010* are displayed by JBrowse (Fig. 3a). The results are organized at three levels, the gene, transcript, and isoform levels. At the gene level, we list the basic information of the gene and its homologs in the two cucumbers (Fig. 3b). At the transcript level, the transcript expression abundance, predicted AS events, and PSI values of these events are reported for each query gene among tissues. This is also illustrated in Fig. 3c, in which a SE event is detected for *Csa5G176010*. The two transcripts are expressed in all the tissues. At the isoform level, the isoform functional annotations (GO annotation and KEGG pathway annotation) and features of the gene isoforms are provided. As shown in Fig. 3d, the two isoforms of *Csa5G176010* present some different functions, such as “binding” and “AMP salvage”.

**Fig. 4:**
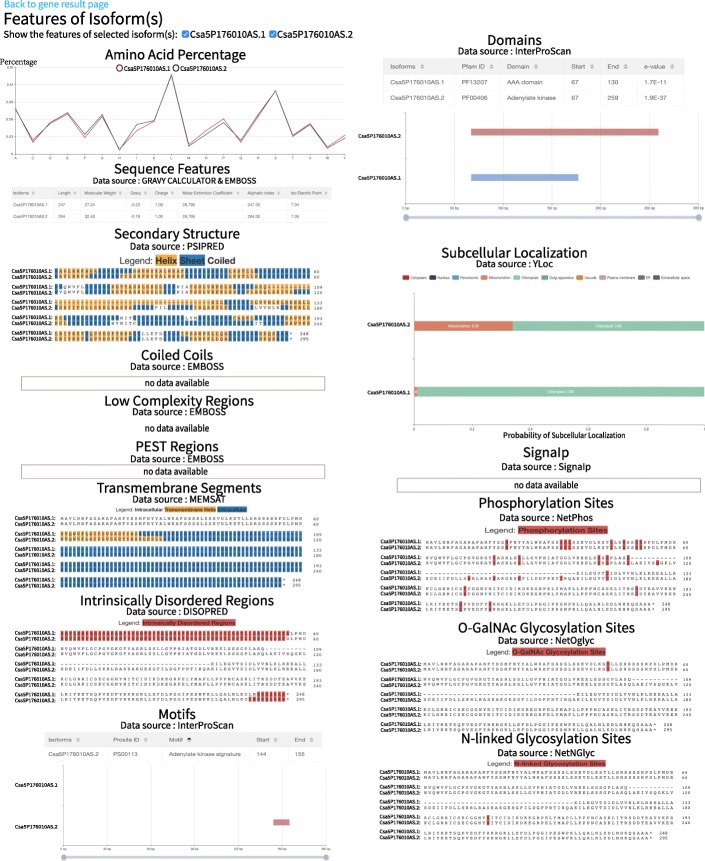
Isoform features of CuAS

The features of the alternative isoforms can be retained by clicking “Show the Features of Isoform(s)” (Fig. 3d). The list of features is shown on the isoform feature page (Fig. 4), including the amino acid composition, sequence features, transmembrane segments, secondary structure, regions of intrinsic disorder, signal peptides, subcellular localization, PEST regions, low-complexity regions, coiled coils, phosphorylation sites, N-linked glycosylation sites, O-GaINAc glycosylation sites, domains, and motifs (see Construction and Content). As shown in Fig. 4 using *Csa5G176010* as an example, Csa5P176010AS.1 includes the “Adenylate kinase signature” motif, but Csa5P176010AS.2 does not include the motif. In addition, there are different functional characteristics between the two transcripts. These results suggest that the SE event detected in *Csa5G176010* has an influence on the function of isoforms.

In addition, two tools are provided: BLAST and JBrowse. BLAST is used to find the homologous sequences of cucumbers. Users can paste their DNA or protein query sequences in the “Query Sequence” box. Users can set search parameters such as the search databases, search programs, maximum number of hits, and E-values. Users can choose the search database by selecting “Searching Against”. Eight BLAST databases including genes, transcripts, CDSs, and isoforms from the two cucumbers were generated for BLAST searches. The search program (BLASTN, TBLASTX, BLASTX, TBLASTN, or BLASTP) can be chosen by selecting “Program”, according to the query sequence and the search database. “Advanced options” can be used to set the maximum number of hits and E-values. JBrowse was applied to visualize the genomic features of cucumbers, including transcripts from multiple cucumber tissues.

Our database offers HTTP links to download the genome sequence, transcript sequences, putative CDSs, and protein sequences in FASTA format. The gene structure annotations can be obtained in the GFF3 format. The list of IDs mapping to UniProt can be obtained. AS events and PSI values can also be downloaded. The list of data files, including isoform features as well as isoform functions, is also accessible in text format. The detailed user manual is available on the CuAS website.

## Conclusions

The advent of RNA-Seq has driven the rapid expansion of transcriptomics. This adds the gap between functional characteristics and transcripts, which is a critical step when trying to understand how diversity may arise from AS. CuAS provides a resource for exploring the relationships between functional features and AS transcripts predicted from multiple tissues in cucumbers, and tissue-specific AS events can be obtained from PSI values. CuAS will help reveal the novel functional features induced by AS and tissue-specific AS events in plants.

CuAS is an ongoing project, and we plan to further develop it in the next release. In particular, we are going to add variation annotations for AS sites and explore the relationship between variation and AS. We also plan to include data related to other organisms, such as *Cucumis melo* L. [37] and *Citrullus lanatus* [38], which will be helpful for achieving a better understanding of AS through comparative analyses in Cucurbitaceae.

## Data Availability

CuAS is freely available at http://cmb.bnu.edu.cn/alt_iso/index.php. The dataset can be downloaded from http://cmb.bnu.edu.cn/alt_iso/index.php/download. The detailed user manual is available at http://cmb.bnu.edu.cn/alt_iso/index.php/help. The website is optimized for Internet Explorer, Mozilla Firefox, Google Chrome, and Safari.

## Abbreviations

A3: alternative 3′ splice-sites
A5: alternative 5′ splice-sites
AS: alternative splicing
CuAS: a database of annotated transcripts generated by alternative splicing in cucumbers
MX: mutually exclusive exons
NMD: nonsense-mediated mRNA decay
ORF: open reading frame
PKs: protein kinases
PSI: percent spliced-in index
RI: retained intron
SE: skipped exon
TFs: transcription factors
TPM: transcripts per million reads
TRs: transcriptional regulators
